# Osmoregulated Chloride Currents in Hemocytes from *Mytilus galloprovincialis*

**DOI:** 10.1371/journal.pone.0167972

**Published:** 2016-12-09

**Authors:** Monica Bregante, Armando Carpaneto, Veronica Piazza, Francesca Sbrana, Massimo Vassalli, Marco Faimali, Franco Gambale

**Affiliations:** 1 Institute of Biophysics, National Research Council of Italy (IBF), Genova, Italy; 2 Institute of Marine Sciences, National Research Council of Italy (ISMAR), Genova, Italy; Tokai University, JAPAN

## Abstract

We investigated the biophysical properties of the transport mediated by ion channels in hemocytes from the hemolymph of the bivalve *Mytilus galloprovincialis*. Besides other transporters, mytilus hemocytes possess a specialized channel sensitive to the osmotic pressure with functional properties similar to those of other transport proteins present in vertebrates. As chloride fluxes may play an important role in the regulation of cell volume in case of modifications of the ionic composition of the external medium, we focused our attention on an inwardly-rectifying voltage-dependent, chloride-selective channel activated by negative membrane potentials and potentiated by the low osmolality of the external solution. The chloride channel was slightly inhibited by micromolar concentrations of zinc chloride in the bath solution, while the antifouling agent zinc pyrithione did not affect the channel conductance at all. This is the first direct electrophysiological characterization of a functional ion channel in ancestral immunocytes of mytilus, which may bring a contribution to the understanding of the response of bivalves to salt and contaminant stresses.

## Introduction

Marine bivalves are common components of the human diet and are also used as bioindicators in environmental monitoring systems. On the other hand, some invertebrates, such as Mediterranean mussels, constitute a major environmental problem for ship hull and industrial power plants that use marine water for cooling; for these reasons *Mytilus galloprovincialis* was inserted as an invasive species in the database of the “100 of the world’s worst invasive species” [1].

Bivalves are able to filter large volumes of seawater, concentrating a series of contaminants within their tissues. They have an open circulatory system, the hemolymph, that is continuously exposed to fluctuating environmental factors, including mechanical and osmotic stresses as well as variable concentrations of salts and contaminants such as inorganic and organic metals. The hemocytes, the circulating cells, are responsible for the immune response of the mytilus to the environment and foreign materials; the roles played by these cells have been suggested to be equivalent to those of monocyte/macrophage lineages in vertebrates. Indeed in *M*. *galloprovincialis* the hemocytes are involved in the phagocytic defence against foreign agents as well as in a series of other physiological functions such as wound and shell repair, nutrient digestion and excretion [2]. Interestingly, it has been reported that bivalves are subjected to disseminated neoplasia and the transmission of independent leukaemia-like diseases within individuals of the same species as well as among different mollusc species [3–6]. Indeed, recent results suggest that the transmission of tumour cells is more frequent in nature than previously thought and therefore studies on bivalves could be of interest also for a better understanding of cancer transmission in general and specifically of metastasization in humans.

The activity of ion channels has been reported in a few tissues of marine mussels; for example the patch-clamp technique was applied to cells from the ventricular myocytes of *Mytilus edulis* in order to characterize some voltage dependent channels [7]: namely two outward potassium currents ascribed to I_k_ and I_A_ channels, an inward L-type calcium channel and a tetrodotoxin sensitive Na-channel.

Despite the rapidly growing body of knowledge on ion transport in immune cells of vertebrates [8–12] as well as molluscs [13–15] little is known on channels in hemocytes of mussels. Only the effects of algal toxins on L-type calcium channels were investigated in the hemocytes from *M*. *galloprovincialis* by immunofluorescence experiments and confocal microscopy [16] or by analysis of cellular parameters and receptor recognition pattern in *Mytilus chilensis* [17].

The capability to respond to anisotonic conditions in these ancient and elementary organisms could be of primary importance to enlarge and improve the knowledge of similar processes in osmoregulated organisms. As it is well known that in mussels the internal medium follows the variations of the osmotic concentrations of the external medium with consequences on the increase/decrease of cellular volume, we adopted the patch-clamp technique in order to verify whether hemocytes under osmotic stress display any mechanism that may contribute to regulate their volume.

In this paper we were able to demonstrate that *Mytilus galloprovincialis* hemocytes possess a variety of specialized channels which appear to be qualitatively similar to other transport molecules previously identified in vertebrates. As chloride channels seem to play a potential role in the regulation of cell volume during transient modifications of the ionic composition of the external medium, we performed experiments in order to characterize the inwardly rectifying anionic current that was present and readily identified in several patch-clamp recordings in *M*. *galloprovincialis* hemocytes. Besides being selective for chloride these channels are voltage-dependent and slowly activated by negative hyperpolarizing membrane potentials in moderate hyposmotic solutions that still allow the organism to activate a reasonable stress response [18]. On the contrary the chloride currents are reversibly inhibited by hyperosmotic conditions. In our working conditions, the currents were slightly inhibited by micromolar ZnCl_2_ concentrations, while the organic compound zinc pyrithione (ZnPT_2_) did not affect at all the current.

## Materials and Methods

The ISMAR marine station was authorized by the Ministry of Infrastructure and Transport through the Genoa Port Authority. We confirm that the field studies did not involve endangered or protected species.

### Hemolymph extraction

**Fig 1:**
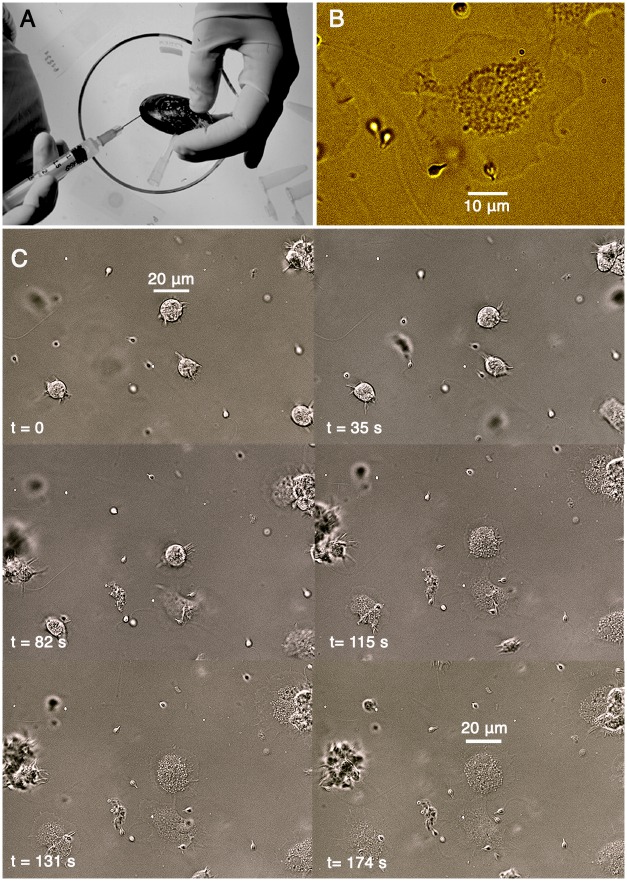
Hemocyte morphology. A) Extraction of the hemolymph from the posterior adductor muscle of *Mytilus galloprovincialis*. B) In a few seconds the hemocytes attached to the bottom of the Petri dish and assumed a very thin and flat shape. The granulocyte lysosomal compartment is easily identifiable in this magnification view (represented in false colours) of a hemocyte firmly attached to the bottom of the recording chamber in Modified Artificial Sea Water (MASW). Note the faint peripheral area comprised between the granule compartment and the cytoplasmic membrane. C) The panel illustrates the progressive flattening and adhesion of granulocytes adhering to the glass bottom of the recording chamber. The process is typically complete in a few minutes. Note the different diameter of the same cell in the first and last frame.

Adult specimens of Mytilus galloprovincialis were collected at the ISMAR marine station and transferred to the lab, cleaned of epibionts, allocated in tanks containing aerated filtered sea water and allowed to acclimate for a few days at 20°C before experiments. The hemolymph was extracted from the posterior adductor muscle using a 2 ml syringe (Fig 1A), put in sterile tubes and kept in ice.

### Microscopy

A home-made opto-electronic platform was used to acquire the optical images. The tool integrates standard modular components (Optem FUSION, Qioptiq Photonics GmbH & Co. KG) and is equipped with a Plan Fluor 40x objective (NIKON Instruments, Amsterdam, The Netherlands) mounted on a motorized Z-axis with a 0.01 μm resolution step. The sample was mounted on a motorized X-Y stage with a 0.5 μm resolution step. A LED lamp and a Gig-E DMK 23G274 camera (The Imaging Source, Bremen, Germany) equipped with a CCD, Sony 1/1.8" (1600x1200 pixels), completed the equipment.

### Cell preparation and electrophysiological recordings

Before each electrophysiology experiment, the hemolymph (osmotic pressure ∏ = 1051±2.5 mosmol/kg) was transferred to a Petri dish (equipped with a glass or plastic bottom) and diluted 1:20 in filtered Modified Artificial Sea Water (MASW) containing (in mM): NaCl 460, KCl 10, MgCl_2_ 2.5, CaCl_2_ 2.5, Hepes 10 at pH = 7.6 with an osmolality ∏ = 867±2 mosmol/kg. The standard pipette solution was (in mM) KCl 530, MgCl_2_ 1, CaCl_2_ 1, EGTA 10, MgATP 2, Hepes 30, pH = 7.5, ∏ = 955 mosmol/kg.

The majority of the patch-clamp experiments were performed in hyposmotic (bath) solution (with low chloride in the bath) containing (in mM): KCl 50, MgCl_2_ 2.5, CaCl_2_ 2.5, LaCl_3_ 0.5, Hepes 10 at pH = 7.6 adjusted to an osmotic pressure ∏ = 897±5 mosmol/kg by the addition of sorbitol.

When needed, the bath solution was adjusted to increase the osmotic pressure to hyperosmotic values by the addition of sorbitol, namely ∏ = 1178±8 mosmol/kg. In the following we indicate this solution (which has the same salt concentrations of the hyposmotic solution) with the term hyperosmotic (bath) solution. To determine the ionic selectivity of the channel under study, when needed, potassium was substituted by N-methyl-D-Glucamine (NMDG) both in the bath and in the pipette solutions.

The osmolality of the solutions was determined with a vapor pressure osmometer 5100C (Wescor Inc., Logan, UT, USA) and the osmolality measurements represent mean values±SEM (n = 20).

The patch-clamp technique [14] was applied to isolated hemocytes in the whole-cell mode. The ionic currents were recorded with an EPC7 (HEKA Instruments) current-voltage amplifier. Data were digitized using a 16 bit Instrutech A/D/A board (Instrutech, Elmont, N.Y.) interfaced to a computer, which generated the voltage stimulation protocol and stored the current responses on the computer hard disk. Current records were low-pass filtered with a 4-pole filter Kemo VBF8 (Kemo, Beckenham UK). When needed, two Ag-AgCl electrodes were supported by agar bridges and the applied voltages were corrected for the appropriate values of the Liquid Junction Potential measured according to [19]. Patch pipettes were pulled from thin-walled borosilicate glass tubing (Clark Electrochemical Instruments, Pangbourne, Reading, UK). The resistance of the patch pipettes in the bathing medium was in the order of 2–4 Mohm.

The ionic selectivity was monitored by the instantaneous values of the deactivating tail currents. A slow or a fast perfusion procedure was adopted to change the solution bathing the cell: in the first case the bath solution was changed by means of a peristaltic pump that was able to renew the entire volume of the bath in a few minutes. Instead in the fast procedure, the bathing medium surrounding the cell was changed using up to five large perfusion pipettes (with a tip in the order of 30 μm) each one filled with a different solution to be investigated [20–22]. Coarse movements were controlled by a hydraulic manipulator that allowed to switch within few seconds between the different perfusion pipettes bathing the cell.

The theoretical reversal potentials for various ions were calculated using the ionic activities coefficients derived from previous papers [23–25]. The activity coefficient of chloride in our experimental conditions (e.g. hyposmotic solution in the bath) was further checked by measuring the equilibrium potential by two Ag-AgCl electrodes (sensitive to chloride) which gave a value of 46.3 mV, in accordance with the theoretical value calculated using the activity coefficients for chloride provided by [25].

The cell capacitance was measured by means of the capacitance compensation circuitry of the voltage amplifier.

The relative open probability of the channel was obtained dividing the steady-state currents by (V-V_Rev_), then the normalized conductance (G_Norm_) was obtained by normalizing to the saturation value, plotted as a function of the driving voltage and best fitted by the Boltzmann distribution for a classical two state model, i.e.:

$$g\;=\;1/\left(1\;+\;exp\left(zF\left(V-V_{1/2}\right)/RT\right)\right)$$ (1)

where F, R and T have the usual meanings, z is the gating charge determining the steepness of the distribution, while V_1/2_ is the half activation potential that depends both on z as well as on non-electrical work required to open the channel [13].

## Results

Isolated hemocytes, displaying a large lysosomal compartment containing many granules, closely resemble (for dimensions and cell morphometric parameters, see Fig 1A–1C) granulocytes already investigated by other authors [2,26–28]. After the transfer to the recording chamber, the cells initially displayed a rounded and ruffled shape, then, in few minutes (i.e. ∼<180 s), the hemocytes typically became very flat, firmly sticking to the bottom of the recording chamber (Fig 1B and 1C). Moreover, minor modifications of the cell shape could be further observed with time, thus suggesting that other molecular mechanisms activate after the transfer to the bath solution.

We verified that the cell population did not show significant qualitative morphometric differences as well as adhesion properties on glass or plastic surface either in the hemolymph itself or in MASW: the increase of the two dimensional area of the cell was typically compensated by a decrease of the thickness which in many cases was reduced in the order of one micron or less (as qualitatively evaluated by our home-made opto-electronic platform). Despite the difficulties in performing patch-clamp experiments on these very flat cells, we were able to perform 94 electrophysiological recordings on mytilus hemocytes.

### Basic electrophysiology

For electrophysiology experiments the hemocytes were typically first diluted in MASW then, when needed, the solution bathing the cell was changed by the slow or the fast perfusion procedure (see Materials and methods). In MASW we readily observed large time-dependent inward currents present mainly at negative membrane potentials (S1A Fig). These inward currents were frequently superimposed to other time-dependent components, such as the K^+^ currents illustrated in S1B Fig This is not surprising as whole-cell currents typically comprise contributions mediated by different channels/transporters. Furthemore time-independent unspecific currents increasing linearly with the applied potential and overwhelming the signals of endogenous channels were occasionally observed.

Osmotic gradients may lead to alterations of the cell volume and to variations of water fluxes through the plasma membrane of hemocytes [27]. Furthermore the osmotic pressure of the ionic solutions and the transmembrane potential, two physical parameters important for cell survival, can also be used to separate the contributions to the ionic fluxes by different transporters.

Therefore, we decided to test whether we were able to isolate the hemocyte most significant current component by combining these two parameters, choosing two values of the osmotic pressure larger and smaller with respect to the osmotic pressure of the hemolymph (∏ = 1051, see Materials and methods). Furthemore, in order to acquire information on the ionic species carrying the charge, we further simplified our working conditions by adopting a solution with a low KCl concentration, a condition that favours larger inward currents. This choice also provides information on the selectivity of the channel(s) owing to the concentration gradient present between the bath and the internal pipette solution that, in the whole cell configuration, replaces the internal milieu of the cell.

Finally, on the basis of our previous experience [29,30], in order to reduce the leak-like time-independent current, lanthanum chloride (LaCl_3_) 0.5 mM was added to the bath solutions. This expedient allowed us to obtain high gigaseals, with minimal interference (I_NoLa_/ I_La_ = 0.9 ± 0.1, N = 7, data not shown), if any, on the properties of the endogenous time-dependent currents [29,30]. Therefore the majority of the experiments were done in the presence of 0.5 mM LaCl_3_ in the bath solution.

### Modulation of the inward current by hypotonicity

**Fig 2:**
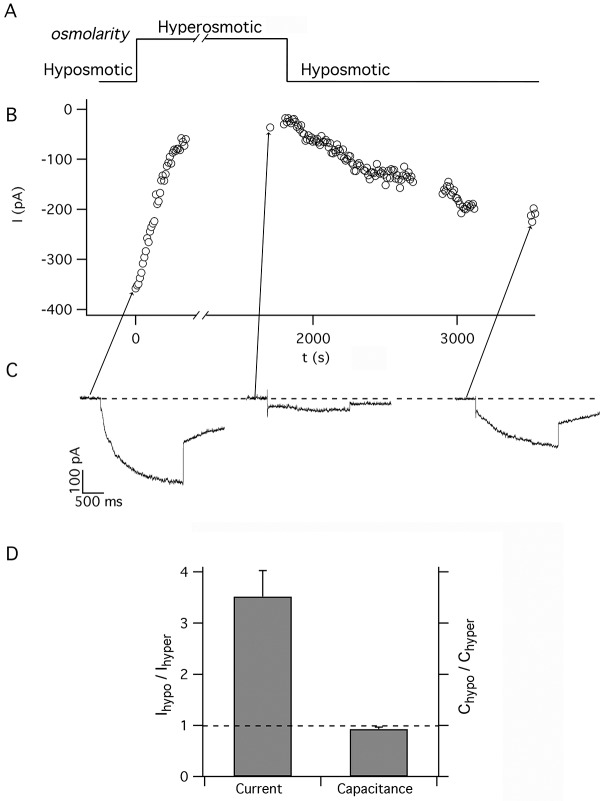
The time-dependent currents in *Mytilus galloprovincialis* hemocytes are modulated by the osmotic pressure. A) Schematic representation of the osmotic pressure of the bath solutions vs time adopted to investigate the response of hemocytes to different osmotic pressures. The osmolality profile shows that the experiment started in the hyposmotic solution (∏ = 897 mosmol/kg), then, at t = 0 s, the bath was perfused with the hyperosmotic solution (∏ = 1178 mosmol/kg) and finally (at t≈1850 s) the bath was again perfused with the hyposmotic solution. B) The mean values of the steady-state sequential currents, elicited every 10 s (see typical currents in panel C), are plotted as a function of time. It can be observed that the increase of the osmolality causes a drastic decrease of the current. The traces in panel C) represent three typical time-dependent currents elicited by voltage pulses to -100 mV (replicated every 10 s) in hyposmotic (1^st^ and 3^rd^ trace, on the left and the right, respectively) and hyperosmotic bath solutions (middle trace). The arrow emerging from each trace points to the correspondent data point in B). D) The hyperosmotic bath solution determines a decrease of the steady-state current elicited by V = -100 mV (left bar) without altering the capacitance of the cells (right bar). Current bar represents the increase of the mean steady-state current in hyposmotic condition with respect to hyperosmotic solution (I_hypo_/I_hyper_ at the left axis) ± SEM from 7 different experiments, while the cell capacitance (C_hypo_/C_hyper_ at the right axis) remained almost unaltered, thus indicating that the current increase is not due to an increase of the cell surface (and to a consequent increase of the number of available channels) in hyposmotic conditions.

**Fig 3:**
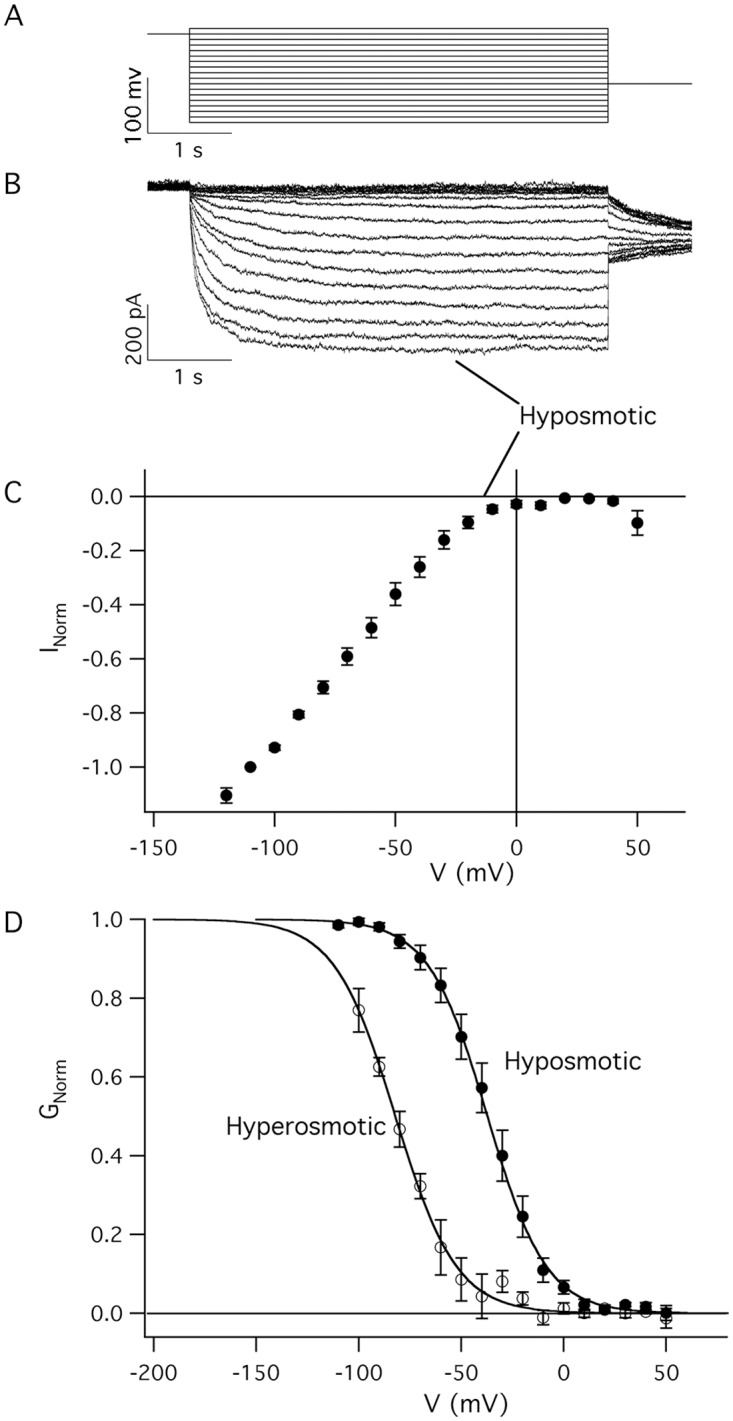
Characteristics of the inwardly-rectifying time-dependent currents in mytilus hemocytes. A) Voltage protocol applied to *M*. *galloprovincialis* hemocytes eliciting time-dependent currents. The duration of the main pulse was 5 s and the applied voltages ranged from +50 mV up to –120 mV in –10 mV decrements. B) Typical whole-cell inwardly rectifying time-dependent currents that activate slowly on the application of the hyperpolarizing pulses represented in A and deactivate in a fraction of seconds after the voltage repolarization to positive values. Holding and tail membrane potentials were +40 mV and -50 mV, respectively. Experiments performed in the hyposmotic bath solution with osmolality equal to 897 mosmol/kg. C) Values of the normalized currents (I_Norm_) obtained mediating the final segment of each steady-state current from at least 4 different experiments. The data were normalized with respect to the absolute value of the current at V = -110 mV and plotted as a function of the applied transmembrane potential. D) Filled symbols represent G_Norm_ in hyposmotic solutions plotted as a function of the membrane potential; the solid line represents the best fit of the experimental data by the Boltzmann equation. Interestingly, in the same set of cells, a comparison of the Boltzmann distributions was left-shifted towards more negative membrane potentials by as much as -44.9 ± 4.4 mV when the hyposmotic solution (filled circles) bathing the cell was replaced by an identical hyperosmotic solution (empty symbols at ∏ = 1178 mosmol/kg), thus implying that more negative membrane potentials are needed to activate the same current at higher osmolality. V_1/2_*(hypo)* = -37.2 ± 1.3 mV and V_1/2_*(hyper)* = -82.1± 3.1 mV, z*(hypo)* = 1.8± 0.1 and (z*(hyper)* = 1.7±0.1).

These conditions provided the typical current traces activated by V = -100 mV in hyposmotic (osmolality ∏ = 897 mosmol/kg) and hyperosmotic (∏ = 1178 mosmol/kg) conditions (see the profile in Fig 2A). The currents displayed in Fig 2C can be compared with a complete current family activated by hyposmotic conditions at different membrane potentials (see Fig 3B).

Indeed in the hyposmotic bath solution we could measure significantly larger time-dependent currents, with respect to the currents observed in the hyperosmotic solution. The current increase induced by the low osmotic pressure was reversible (as illustrated by the typical traces in Fig 2C) and by the plot of the steady-state current at V = -100 mV (Fig 2B) at the two different osmolalities. In order to verify whether the current increase in hyposmotic solution could be ascribed to any change in the membrane surface of the cells, we simultaneously monitored both the currents and the capacitances of the hemocytes in hyposmotic and hyperosmotic conditions (Fig 2D). Interestingly while in hyposmotic solution the mean current was 3.5 times the value measured in hyperosmotic solution, the cell capacitance remained unaffected, thus indicating that the increase of the current could not be ascribed to an increase of the membrane surface and therefore to a recruitment of new channels by the fusion of endocytotic vesicles.

### Voltage dependence of the inward current

**Fig 4:**
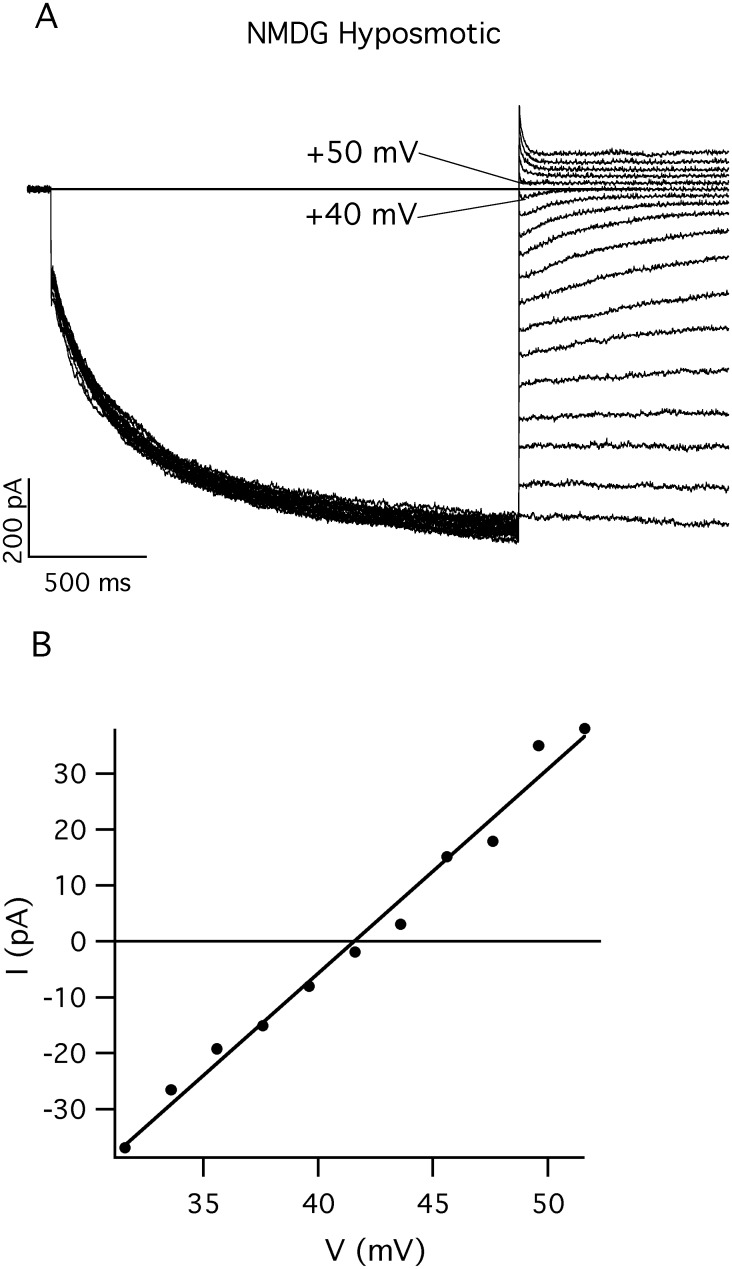
The time dependent currents are mediated by chloride channels. A) Tail currents elicited by voltages ranging from -80 mV to + 90 mV in 10 mV steps after a main pulse to -80 mV from a holding potential of +40 mV. In the standard pipette solution and hyposmotic bath solution, NMDG-chloride replaced 530 mM and 50 mM KCl, respectively. Clearly the tail currents inverted at potentials comprised between V = +40 mV and V = +50 mV (indicated by the two lines), i.e. at a value compatible with the Nernst potential for chloride in this working conditions (V_Nernst_(Cl^-^) = +46 mV). B) Instantaneous values of the tail currents (extrapolated at t = 0 s) are plotted as a function of the tail potentials in the range from +32 mV to +52 mV, incremented by 2 mV steps after the main pulse to -80 mV.

In hyposmotic bath solution the slow inwardly-rectifying currents were typically elicited by hyperpolarizing pulses (see the voltage protocol in Fig 3A), activated slowly in a time-dependent manner (Fig 3B) and reached a steady-state plateau in a time lapse (dependent on the applied voltage) in the order of hundreds milliseconds. Finally these currents deactivated at repolarizing membrane potentials (see the tail currents in Figs 3B and 4A).

In order to quantify the dispersion of the current, the mean values of the steady-state currents were normalized to the steady-state current at V = -110 mV and the normalized current (I_Norm_) was plotted as a function of the applied membrane potential (Fig 3C). From these data we could calculate the normalized conductance (G_Norm_ in Fig 3D). In hyposmotic solutions, G_Norm_ increased as a function of the membrane potential, with the tendency to saturate at large negative membrane potentials, as displayed in Fig 3D, where the Boltzmann distribution (continuous line) obtained from the best fit of the experimental data (filled symbols) is reported as a function of the applied potential. The gating charge z and the half activation potential (see Materials and methods) were: z*(hypo)* = 1.8± 0.1 and V_1/2_*(hypo)* = -37.2 ± 1.3 mV (n = 11).

The comparison of the Boltzmann distributions (Fig 3D) provides further support to the hypothesis that the activation of the inward current was favoured by the hypotonicity of the bath solution. Indeed the current decrease induced by the hyperosmotic solution is clearly due to a shift of the half activation potential of the current towards more negative membrane potentials: the activation potential of the Boltzmann equation was shifted by about-45 mV (z*(hyper)* = 1.7±0.1 and V_1/2_*(hyper)* = -82.1± 3.1 mV), towards more negative potentials changing the bath solution from the hyposmotic solution (filled symbols in Fig 3D) to hyperosmotic solution (empty symbols in Fig 3D).

### The inward rectifying current is mediated by a chloride selective channel

Other evidences demonstrate that the inward currents were mediated by a chloride selective channel. Indeed, experiments performed in the absence of K^+^ (i.e. substituting NMDG in the bath and in the pipette solutions, Fig 4A and 4B), or in MASW bath solution (S2A and S2B Fig) or in the hyposmotic bath solution (in both cases with standard pipette solution in the pipette) (S2C Fig) demonstrated that neither K^+^ nor Na^+^ could be responsible for the ionic currents mediated by the inwardly rectifying channel. Indeed, the reversal potentials (V_Rev_) under diverse conditions, were always in accordance with a chloride selective channel. Furthermore, under a stimulation protocol applied to elicit a series of time-dependent currents (at V = -80 mV) followed by tail currents ranging from -80 mV up to +90 mV, in NMDG-Cl 50 mM in the bath and 530 mM NMDG-Cl in the pipette, V_Rev_ was clearly comprised between +40 mV and +50 mV, as indicated by the two lines in Fig 4A and by the plot of the instantaneous tail currents vs. the tail potential in Fig 4B. Finally, in NMDG we were able to measure a mean reversal potential (after the correction for the Liquid Junction Potential) equal to V_Rev_ = +40±2 mV (n = 8) at pH 7.6 as well as V_Rev_ = +42±4 mV (n = 3) at pH 6.0: two values which are very close to what expected for a chloride-selective channel (V_Nernst_(Cl^-^) = +46 mV).

In MASW (S2B Fig) the tail currents (see the stimulation protocol in S2A Fig) inverted at slightly positive voltages (V_Rev_ comprised between 0 mV and +10 mV as indicated by the two lines reported in panel B), a value that also in this case is in accordance with the Nernst potential for chloride (V_Nernst_(Cl^-^) = +2 mV) and very far from the Nernst potentials for potassium (V_Nernst_(K^+^) = -97 mV) and sodium which would be positive and indefinitely large.

Consistently with the value measured in the presence of internal and external NMDG, also in the hyposmotic KCl bath solution (S2C Fig), the reversal potential was ≈+ 50 mV a value which was again very close to the Nernst potential for chloride (V_Nernst_(Cl^-^) = +46 mV) and very distant from the Nernst potential for potassium (V_Nernst_(K^+^) = -53 mV).

All these data consistently confirmed that potassium and sodium did not contribute to the time-dependent hemocyte currents. As some chloride transport proteins are chloride/proton antiporters, in order to verify the nature of the hemocyte chloride transporter, we also changed the pH of the hyposmotic bath solution: in the pH range from 6.0 to 8.0 (data not shown) we did not observe any variation of the reversal potential, thus providing a confirmation that the time-dependent currents were mediated by a chloride selective channel and not by a chloride/proton antiporter [31,32].

Finally, when we replaced NaCl of the external MASW with an identical concentration of NaGluconate, a larger negative current was observed (S3 Fig). Consistently with other CLC-2 channels, this can be explained by the lower permeability of gluconate [33] with respect to chloride through the channel. The lower gluconate permeability shifts the reversal potential (from a few mV, see S2B Fig) towards more positive values, determining a greater driving force and a consequent larger chloride current flowing out of the cell.

### Effects of zinc and zinc pyrithione on the inward rectifying channel

**Fig 5:**
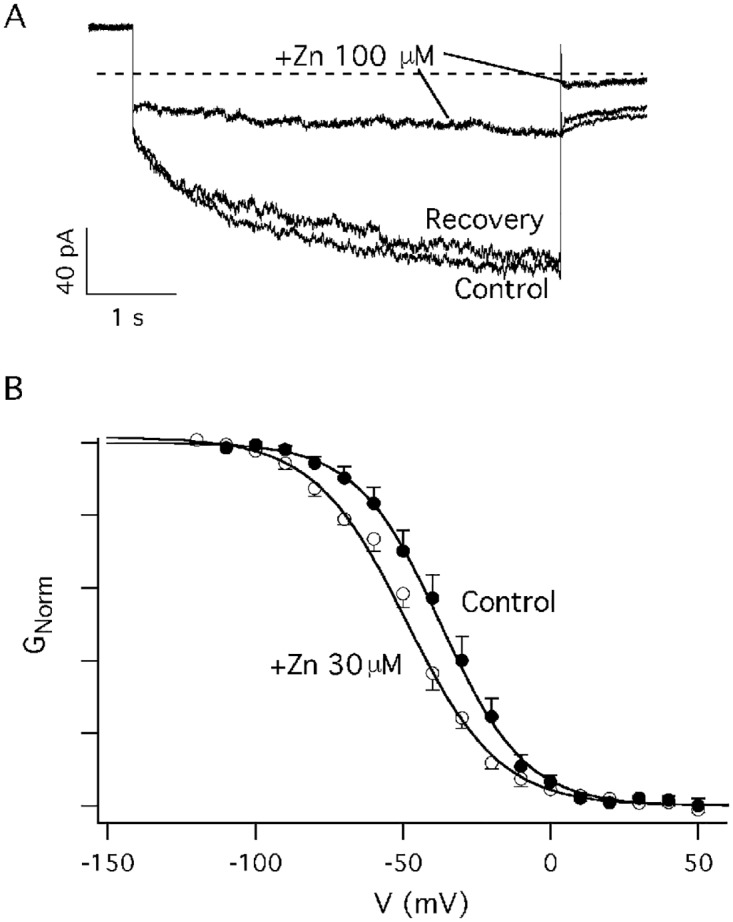
Micromolar ZnCl_2_ reduces the amplitude of the time-dependent current. A) The decrease of a typical inward time-dependent current induced by the addition of 100 μM ZnCl_2_ to the bath solution. A series of step voltages to -120 mV were applied to the cell with an interval of 15 s in the presence and in the absence of 100 μM ZnCl_2_; the holding potential was +20 mV, tail voltage was -50 mV. Control and recovery: hyposmotic solution. Each current trace represents the average of at least 3 different traces obtained in the same conditions. B) The Boltzmann distribution is shifted towards more negative membrane potentials (ΔV = -10.3 ± 2.3 mV) on the addition of 30 μM ZnCl_2_ (empty circles) to the bath solution with respect to the control conditions (filled circles, hyposmotic bath solution). It can be observed that the current decrease can be ascribed to a shift to the left of the Boltzmann distribution. Data were obtained averaging at least 4 different current records in the different conditions. V_1/2_*(hypo)* = -37.2 ± 1.3 mV and V_1/2_*(Zn*^*2+*^ *= 30 mM)* = -47.5± 1.0 mV, z*(hypo)* = 1.8 ± 0.1 and z*(Zn*^*2+*^ *= 30 mM)* = 1.7± 0.1.

Looking for ions that may interact with the inwardly rectifying channel of mussel hemocytes, we verified that the addition of zinc to the bath solution determined a decrease of the current. Fig 5A displays the typical current decrease induced by 100 μM ZnCl_2_ on currents elicited by a voltage pulse to -120 mV, while Fig 5B displays the Boltzmann distribution of the normalized conductance recorded in the absence and in the presence of 30 μM ZnCl_2_. Zinc addition to the bath solution induced a smaller reduction of the chloride current but qualitatively similar to what induced by the increase of the bath osmolality: i.e. we measured a shift of V_1/2_ towards more negative potentials equal to ΔV_1/2_ = -10.3 ±2.3 mV (z*(Zn*^*2+*^ *= 30 mM)* = 1.7 ± 0.1 and V_1/2_*(Zn*^*2+*^ *= 30 mM)* = -47.5±1 mV from at least 4 different experiments).

In order to verify whether the effects of zinc depend on the ionic form of the metal, we also tested zinc pyrithione, a well-known antifouling, antifungal and antibacterial agent where zinc is bound to two sulphur and two oxygen atoms. However, up to 30 μM ZnPT_2_ did not affect the current appreciably: i.e. I_ZnPT2_/I_control_ = 1.1 ± 0.1 (n = 4, data not shown).

## Discussion

Bivalve granulocytes are characterized by a large number of electron-dense internal granules: it has been reported that they represent the major population of cells present in the hemolymph of *M*. *galloprovincialis* [34]. In our experimental conditions, when the hemolymph was transferred to the petri dish chamber for the electrophysiological characterization, the hemocytes readily assumed a very flat configuration that made difficult to identify any distinct morphological characteristic. In addition, the mytilus hemocytes investigated by electrophysiological means displayed a series of different current components but no significant differences that might suggest the existence of different populations.

### Regulatory volume decrease

Regulatory Volume Decrease (RVD) is generally achieved by the loss of ions and other osmolytes and the concomitant loss of water that is regulated by the transport of ions and/or osmolytes through the plasma membrane and the subsequent water efflux out of the cell. In many cell types, including bivalves [35], this behaviour is typically mediated by potassium and chloride electroneutral co-transport as well as VRAC (Volume-Regulated Anion Channels) which are typically inactive under resting conditions, but are able to contribute to a partial recovery of the cell size by a regulatory volume decrease mechanism in cells subjected to hypotonic stress [36–38].

It has been shown that mussels are sensitive to the chloride concentration of the bathing medium. In general, bivalves are able to survive to water chlorination by adopting defence strategies that induce the mussel to shut their valves as soon as they detect an anomalous chloride concentration [39,40]. It is well known that in molluscs the osmotic concentration of the internal medium follows the variations of the external environment and the hypotonic stress determines the swelling of diverse cell types, followed by the recovery of the original volume. For example, by using videometric methods it has been demonstrated that the cells from the digestive glands *of M*. *galloprovincialis*, exposed to rapid changes of the bathing solution (from 1100 to 800 mosmol/kg), undergo to a process of regulatory volume decrease [35]. Possibly the minor movements of *M*. *galloprovincialis* hemocytes (observed after their adhesion to the glass bottom) may depend on rearrangements due to a slow cell shrinkage (driven by the hypotonicity) that follows the faster reactions induced in the cells that perceive to be in a medium of different composition with respect to the hemolymph.

### Ionic currents in mussel hemocytes

In mytilus hemocytes, beside the inward-rectifying channel, we also recognized a time- and a voltage-independent current component (increasing linearly with the voltage, data not shown) and occasional typical K^+^ outward currents (S1B Fig) which could be ascribed to an n-type inactivating potassium channel [41,42] that looks very similar to animal potassium channel recorded, for example, in rat thymocytes [9].

A large number of different types of chloride channels such as cAMP-, calcium-, ligand- and voltage-gated channels as well as volume regulated chloride channels are expressed almost ubiquitously in plant and animal tissues [43–45]. The kinetics and characteristics of the hemocyte inward channel are strongly reminiscent of the properties of the slow-activating volume-regulated chloride channel CLC-2 [38,45–48]. This channel type is broadly expressed in a variety of vertebrate tissues—including brain, kidney, liver and heart—and cells—from epithelia to neurons. CLC-2 is inactive under basal physiological voltages and therefore it is supposed to be regulated by a series of parameters, such as the cell swelling [45]. Like in animal ClC-2 channel, in our working conditions, also the inward hemocyte channel displayed a strong dependence on the osmolality of the bathing solution.

### Biophysical characteristics of the currents

The activation properties of the inward rectifying channel are well represented by the Boltzmann distribution that characterizes the normalized macroscopic conductance as a function of the applied potential and which provides information on the work to be done to open a voltage-dependent channel. Clearly hyposmotic bath solutions contribute to shift the range of activation (well represented by V_1/2_) of the hemocyte inward-rectifying channel towards more positive membrane potentials. Interestingly, the steepness of the Boltzmann distribution does not change appreciably in Fig 3, thus indicating that the charges involved in the gating of the channel were not affected by the osmolality of the external solution. Consequently, the work required to open the channel seems to depend on additional non-electrical work that needs to be done in hyperosmotic conditions. Thus indicating a lower mobility of an uncharged segment of the protein which possibly plays a role in the channel opening.

### Biocides

Copper and zinc are important contaminants of the marine environment owing to the large use of these metals as antifouling agents: zinc is frequently used as a weak primary biocidal pigment, but it is also adopted in combination with copper as a booster that increases hundreds fold the toxicity of Cu. As some organisms are resistant to inorganic metals, other agents, such as zinc pyrithione or copper pyrithione, are added as co-biocides to antifouling blends [49].

In addition, chemical modulators are useful tools to investigate the properties of ion channels. As it was demonstrated that ZnCl_2_ and ZnPT_2_ are able to modulate the activities of native and expressed ion channels [50–52], we verified whether the addition of micromolar concentrations of these two zinc compounds may have any effect on the osmoregulated chloride channel in hemocytes. On the addition of ZnCl_2_ to the bath solution, in hyposmotic conditions one can observe a shift of the Boltzmann distribution towards negative membrane potentials. With respect to the control, this shift is definitely smaller but still appreciable compared to the shift observed on hyperosmotic conditions (see Fig 5). Interestingly, also in this case "z" remained almost unaltered (i.e. between 1.7 and 1.8 charge units, see the values reported in the legends of Figs 3 and 5). Incidentally, one can also observe that the decrease of the current induced by 30 μM Zn^2+^ on the osmoregulated channel is in accordance with a comparable decrease of the CLC-2 chloride current which was reported to occur in native hyppocampal pyramidal cells that naturally express CLC-2 as well as in dorsal root ganglion cells overexpressing exogenous CLC-2 [53,54]. Interestingly, Zn^2+^ also inhibits other chloride channels and transporters, such as ClC-1 and ClC-4 [45].

The observation that ZnPT_2_ did not affect at all the chloride current of hemocytes possibly depends on the fact that pyrithione ligands are formally monoanions that chelate Zn^2+^ via oxygen and sulfur centers. The pyrithione speciation with metals is relatively strong both in fresh and marine water and dissociation time is in the order of days (in the absence of light) while several hours are necessary under photolysis conditions [55–57]. Therefore we expect that during a typical patch-clamp experiment, ZnPT_2_ remains almost unaltered. The fact that zinc pyrithione did not affect the conductance of the channel suggests that zinc must be in its divalent ionic form to be effective on the hypotonicity activated channel. However, the Boltzmann distribution (Fig 5) also suggests that the effect of Zn^2+^ does not depend on a modification of the gating charge of the channel: possibly other mechanisms depending on the ionic charge of the metal could reduce the mobility of specific segments of the channel. In alternative Zn^2+^, but not ZnPT_2_ may change some properties of the lipids surrounding the protein, that in turn might affect the channel properties [58].

## Conclusions

Of the various systems that can contribute to RVD, swelling-activated chloride channels are present in a number of cell types. It has been shown that many cells and, among them, immunocells are able to slowly down regulate their volume after a rapid swelling under hyposmotic conditions [59]. It is well recognized that potassium and chloride transport [38] typically contribute to RVD in several cell types and specifically in immunocells, such as thymocytes from rats [59] and mice [60] as well as lymphoblastic leukemia cells [44]. Interestingly it has also been suggested that in *Mytilus galloprovincialis* digestive cells [35] and in *Mytilus californianus* gill cells [61] K^+^ and Cl^_^ cooperate in RVD by the efflux of these two ions followed by an obliged efflux of water from the cell.

Owing to hypotonicity and voltage dependence of the hemocyte inwardly-rectifying current, we argue that, under basal physiological conditions, similarly to other channels involved in RVD [36–38] the inward channel has very small activity if any. Instead, it might be activated by a decrease of the osmolality of the external solution and/or by hyperpolarization: for example, hypotonic conditions could be determined by a dilution of sea water during heavy rainfall, river run off, climatic changes affecting the ocean conveyor belt. These processes may determine local and temporary decrease of water salts mainly at the sea surface. Since molluscs are osmoconformers [35], after a hypotonic water dilution the decrease of environmental [K^+^] as well as other parameters and osmolytes [38] could determine a transient hyperpolarization of the cell and a simultaneous activation of inward chloride currents, i.e. outward chloride fluxes. In turn, this would induce a successive depolarisation and a parallel export of K^+^, Na^+^ [62] and other organic compounds [38,61,63]. The net release of chloride and other ions as well as small organic molecules from the cell will contribute to partially counteract the osmotic stress avoiding a damage of the membrane due the fast cell swelling.

Furthermore, these mechanisms could allow the hemocytes to alert the organism that the external conditions are changing. Some compounds, such as ZnCl_2_ but not ZnPt_2_, may interfere with these signals impairing the immunological response of the organism in critical conditions.

## Supporting Information

S1 Fig Macroscopic currents in *Mytilus galloprovincialis* hemocytes. A) Lower panel: inward slowly activating currents with a slight time independent components are activated in *M*. *galloprovincialis* hemocytes by the voltage protocol illustrated in the upper panel, showing 5 s stimulation steps ranging from +50 mV up to –100 mV in –10 mV decrements. Holding and tail membrane potentials were +40 mV. B) Lower panel: macroscopic currents mediated by an outwardly rectifying channel in the hyperosmotic bath solution (i.e. 50 mM internal K^+^, ∏ = 1178 mosmol/kg) and standard pipette solution. Voltage pulses ranged from -50 mV to +100 mV in 10 mV increments. Holding and tail potentials were -80 mV and +20 mv, respectively. Currents were corrected for leakage.
(TIF)

S2 Fig Selectivity properties of the inward rectifying current. A) Voltage protocol applied to reveal the tail currents in MASW. B) In MASW tail currents of the inward channel inverted at a potential (indicated by the arrow) comprised between 0 and 10 mV, a value compatible with the Nernst potential for chloride (V_Nernst_(Cl^-^) = +2mV) and very different from the Nernst potential for potassium (V_Nernst_(K^+^) = -97mV). Standard pipette solution. C) Tail currents obtained in hyposmotic KCl standard solution. Tail voltages (indicated at the left side of the plot) ranged from 0 mV to + 70 mV. Also in this case a reversal potential of about +50 mV (indicated by the arrow) is in good agreement with the Nernst potential for chloride (V_Nernst_(Cl^-^) = +46 mV) and very different from (V_Nernst_(K^+^) = -53 mV).
(TIF)

S3 Fig Gluconate is less permeable than chloride through the inward-rectifying channel. Currents recorded in MASW and in an identical solution where 460 mM NaCl in the bath was substituted by Na-Gluconate. Currents were elicited by a main pulse to -100 mV from a holding and tail voltages at V = +40 mV.
(TIF)

## References

[1] Lowe S., Browne M., Boudjelas S., De Poorter M. 100 of the World’s worst invasive alien species a selection from the global invasive species database In: Aliens. Published by The Invasive Species Specialist Group (ISSG) a specialist group of the Species Survival Commission (SSC) of the World Conservation Union (IUCN); 2000.

[2] Ottaviani E. Immunocyte: the invertebrate counterpart of the vertebrate macrophage. Inv Surv J. 2011;8:1–4.

[3] Barber BJ. Neoplastic diseases of commercially important marine bivalves. Aquat Living Resour. 2004;17(4):449–66.

[4] Metzger MJ, Villalba A, Carballal MJ, Iglesias D, Sherry J, Reinisch C, et al Widespread transmission of independent cancer lineages within multiple bivalve species. Nature. 2016 6 30;534(7609):705–9. 10.1038/nature18599 27338791 PMC4939143

[5] Metzger MJ, Reinisch C, Sherry J, Goff SP. Horizontal transmission of clonal cancer cells causes leukemia in soft-shell clams. Cell. 2015 4;161(2):255–63. 10.1016/j.cell.2015.02.042 25860608 PMC4393529

[6] Murchison EP. Cancer: Transmissible tumours under the sea. Nature. 2016 6 30;534(7609):628–9. 10.1038/nature18455 27350242

[7] Curtis T, Depledge M, Williamson R. Voltage-activated currents in cardiac myocytes of the blue mussel, *Mytilus edulis*. Comp Biochem Physiol A Mol Integr Physiol. 1999 10;124(2):231–41.

[8] Chandy KG, DeCoursey TE, Cahalan MD, McLaughlin C, Gupta S. Voltage-gated potassium channels are required for human T lymphocyte activation. J Exp Med. 1984 8 1;160(2):369–85. 6088661 10.1084/jem.160.2.369 PMC2187449

[9] Fiorica-Howels E, Gambale F, Horn R, Osses L, Spector S. Phencyclidine blocks voltage-dependent potassium currents in murine thymocytes. J Pharmacol Exp Ther. 1990;252:610–5. 2313591

[10] Hansen LK. The role of T cell potassium channels, KV1.3 and KCa3.1, in the inflammatory cascade in ulcerative colitis. Dan Med J. 2014 11;61(11):B4946 25370966

[11] Bose T, Cieślar-Pobuda A, Wiechec E. Role of ion channels in regulating Ca^2+^ homeostasis during the interplay between immune and cancer cells. Cell Death Dis. 2015;6:e1648 10.1038/cddis.2015.23 25695601 PMC4669790

[12] Feske S, Wulff H, Skolnik EY. Ion channels in innate and adaptive immunity. Annu Rev Immunol. 2015 3 21;33(1):291–353. 25861976 10.1146/annurev-immunol-032414-112212 PMC4822408

[13] Hille B. Ionic channels of excitable membrane. Third Sunderland, MA: Sinauer Ass. Inc; 2001.

[14] Hamill OP, Marty A, Neher E, Sakmann B, Sigworth FJ. Improved patch-clamp techniques for high-resolution current recording from cell and cell-free membrane patches. Pfluƒgers Arch. 1981;391:85–100. 10.1007/BF00656997 6270629

[15] Kodirov SA. The neuronal control of cardiac functions in Molluscs. Comp Biochem Physiol A Mol Integr Physiol. 2011 10;160(2):102–16. 10.1016/j.cbpa.2011.06.014 21736949 PMC5480900

[16] Malagoli D, Casarini L, Ottaviani E. Algal toxin yessotoxin signalling pathways involve immunocyte mussel calcium channels. Cell Biol Int. 2006 9;30(9):721–6. 10.1016/j.cellbi.2006.05.003 16787753

[17] Astuya A, Carrera C, Ulloa V, Aballay A, Núñez-Acuña G, Hégaret H, et al Saxitoxin modulates immunological parameters and gene transcription in *Mytilus chilensis* hemocytes. Int J Mol Sci. 2015;16(7):15235–50. 10.3390/ijms160715235 26154765 PMC4519897

[18] Lockwood BL, Somero GN. Transcriptomic responses to salinity stress in invasive and native blue mussels (genus *Mytilus*): transcriptomes of invasive vs. native mussels. Mol Ecol. 2011 2;20(3):517–29. 10.1111/j.1365-294X.2010.04973.x 21199031

[19] Neher E. Correction for liquid junction potentials in patch clamp experiments. Methods Enzym. 1992;207:123–31. 10.1016/0076-6879(92)07008-c 1528115

[20] Gambale F, Bregante M, Stragapede F, Cantu’ AM. Ionic channels of the sugar beet tonoplast are regulated by a multi-ion single-file permeation mechanism. J Membr Biol. 1996;154:69–79. 8881028 10.1007/s002329900133

[21] Carpaneto A, Cantù AM, Gambale F. Redox agents regulate ion channel activity in vacuoles from higher plant cells. FEBS Lett. 1999;442:129–32. 9928987 10.1016/s0014-5793(98)01642-1

[22] Carpaneto A, Cantù AM, Gambale F. Effects of cytoplasmic Mg^2+^ on slowly activating channels in isolated vacuoles of *Beta vulgaris*. Planta. 2001;213:457–68. 11506369 10.1007/s004250100519

[23] Robinson RA, Stokes RH. Electrolyte solutions. Butterworths Scientific Publications; 1959.

[24] Hamer WJ, Wu Y-C. Osmotic coefficients and mean activity coefficients of monovalent electrolytes in water at 25°C. J Phys Chem. 1972;1(4):1047–100.

[25] Dash D, Kumar S, Mallika C, Mudali UK. New data on activity coefficients of potassium, nitrate, and chloride ions in aqueous solutions of KNO _**3**_ and KCl by ion selective electrodes. ISRN Chem Eng. 2012;2012:1–5.

[26] Ottaviani E, Franchini A, Barbieri D, Kletsas D. Comparative and morphofunctional studies on *Mytilus galloprovincialis* haemocytes: presence of two aging-related haemocyte stages. Ital J Zool. 1998;65:149–354.

[27] Calisi A, Lionetto MG, Caricato R, Giordano ME, Schettino T. Morphometric alterations in *Mytilus galloprovincialis* granulocytes: a new biomarker. Environ Toxicol Chem. 2008;27(6):1435–41. 10.1897/07-396 18260695

[28] Le Foll F, Rioult D, Boussa S, Pasquier J, Dagher Z, Leboulenger F. Characterisation of *Mytilus edulis* hemocyte subpopulations by single cell time-lapse motility imaging. Fish Shellfish Immunol. 2010 2;28(2):372–86. 10.1016/j.fsi.2009.11.011 19944763

[29] Bregante M, Carpaneto A, Pastorino F, Gambale F. Effects of mono- and multi-valent cations on the inward-rectifying potassium channel in isolated protoplasts from maize roots. Eur Biophys J. 1997;26:381–91.

[30] Carpaneto A, Magrassi R, Zocchi E, Cerrano C, Usai C. Patch-clamp recordings in isolated sponge cells (*Axinella polypoides*). J Biochem Biophys Methods. 2003;55(2):175–85. 10.1016/s0165-022x(02)00184-7 12628699

[31] Accardi A, Miller C. Secondary active transport mediated by a prokaryotic homologue of ClC Cl- channels. Nature. 2004;427(6977):803–7. 10.1038/nature02314 14985752

[32] De Angeli A, Monachello D, Ephritikhine G, Frachisse JM, Thomine S, Gambale F, et al The nitrate/proton antiporter AtCLCa mediates nitrate accumulation in plant vacuoles. Nature. 2006;442:939–42. 10.1038/nature05013 16878138

[33] De Jesús-Pérez JJ, Castro-Chong A, Shieh R-C, Hernández-Carballo CY, De Santiago-Castillo JA, Arreola J. Gating the glutamate gate of CLC-2 chloride channel by pore occupancy. J Gen Physiol. 2016 1;147(1):25–37. 10.1085/jgp.201511424 26666914 PMC4692487

[34] Cajaraville MP, Pal SG. Morphofunctional study of the haemocytes of the bivalve mollusc *Mytilus galloprovincialis* with emphasis on the endolysosomal compartment. Cell Struct Funct. 1995;20(5):355–67. 8581991 10.1247/csf.20.355

[35] Torre A, Trischitta F, Corsaro C, Mallamace D, Faggio C. Digestive cells from *Mytilus galloprovincialis* show a partial regulatory volume decrease following acute hypotonic stress through mechanisms involving inorganic ions: cell volume and mussel digestive gland. Cell Biochem Funct. 2013 8;31(6):489–95. 10.1002/cbf.2925 23112133

[36] Qiu Z, Dubin AE, Mathur J, Tu B, Reddy K, Miraglia LJ, et al SWELL1, a plasma membrane protein, is an essential component of volume-regulated anion channel. Cell. 2014 4 10;157(2):447–58. 10.1016/j.cell.2014.03.024 24725410 PMC4023864

[37] Voss FK, Ullrich F, Münch J, Lazarow K, Lutter D, Mah N, et al Identification of LRRC8 heteromers as an essential component of the volume-regulated anion channel VRAC. Science. 2014 5 9;344(6184):634–8. 10.1126/science.1252826 24790029

[38] Jentsch TJ. VRACs and other ion channels and transporters in the regulation of cell volume and beyond. Nat Rev Mol Cell Biol. 2016;17(5):293–307. 10.1038/nrm.2016.29 27033257

[39] Bott TR. Fouling of heat exchangers. Amsterdam; New York: Elsevier; 1995 524 p. (Chemical engineering monographs).

[40] Rajagopal S, Van der Velde G, Van der Gaag M, Jenner HA. How effective is intermittent chlorination to control adult mussel fouling in cooling water systems? Water Res. 2003 1;37(2):329–38. 12502062 10.1016/s0043-1354(02)00270-1

[41] Zhou M, Morais-Cabral JH, Mann S, MacKinnon R. Potassium channel receptor site for the inactivation gate and quaternary amine inhibitors. Nature. 2001 6 7;411(6838):657–61. 10.1038/35079500 11395760

[42] Lewis RS, Cahalan MD. Subset-specific expression of potassium channels in developing murine T lymphocytes. Science. 1988;239:771–5. 2448877 10.1126/science.2448877

[43] Carpaneto A, Accardi A, Pisciotta M, Gambale F. Chloride channels activated by hypotonicity in N2A neuroblastoma cell line. Exp Brain Res. 1999 1;124:193–9. 9928842 10.1007/s002210050614

[44] Cao G, Zuo W, Fan A, Zhang H, Yang L, Zhu L, et al Volume-sensitive chloride channels are involved in maintenance of basal cell volume in human acute lymphoblastic leukemia cells. J Membr Biol. 2011 3;240(2):111–9. 10.1007/s00232-011-9349-7 21347611

[45] Stauber T, Weinert S, Jentsch TJ. Cell biology and physiology of CLC chloride channels and transporters. Compr Physiol. 2012 7;2(3):1701–44. 10.1002/cphy.c110038 23723021

[46] Jentsch TJ. CLC chloride channels and transporters: from genes to protein structure, pathology and physiology. Crit Rev Biochem Mol Biol. 2008 1;43(1):3–36. 10.1080/10409230701829110 18307107

[47] Lorenz C, Pusch M, Jentsch TJ. Heteromultimeric CLC chloride channels with novel properties. Proc Natl Acad Sci U S A. 1996 11 12;93(23):13362–6. 8917596 10.1073/pnas.93.23.13362 PMC24098

[48] Thiemann A, Gründer S, Pusch M, Jentsch TJ. A chloride channel widely expressed in epithelial and non-epithelial cells. Nature. 1992 3 5;356(6364):57–60. 10.1038/356057a0 1311421

[49] Turner A. Marine pollution from antifouling paint particles. Mar Pollut Bull. 2010 2;60(2):159–71. 10.1016/j.marpolbul.2009.12.004 20060546

[50] Ermolayeva E, Sanders D. Mechanism of pyrithione-induced membrane depolarization in Neurospora crassa. Appl Environ Microbiol. 1995 9;61(9):3385–90. 7574648 10.1128/aem.61.9.3385-3390.1995 PMC167618

[51] Xiong Q, Sun H, Li M. Zinc pyrithione-mediated activation of voltage-gated KCNQ potassium channels rescues epileptogenic mutants. Nat Chem Biol. 2007 5;3(5):287–96. 10.1038/nchembio874 17435769

[52] Wickenden A, McNaughton-Smith G. Kv7 channels as targets for the treatment of pain. Curr Pharm Des. 2009 5 1;15(15):1773–98. 19442190 10.2174/138161209788186326

[53] Staley K, Smith R, Schaack J, Wilcox C, Jentsch TJ. Alteration of GABAA receptor function following gene transfer of the CLC-2 chloride channel. Neuron. 1996 9;17(3):543–51. 8816717 10.1016/s0896-6273(00)80186-5

[54] Clark S, Jordt SE, Jentsch TJ, Mathie A. Characterization of the hyperpolarization-activated chloride current in dissociated rat sympathetic neurons. J Physiol. 1998 2 1;506 (Pt 3):665–78. 9503329 10.1111/j.1469-7793.1998.665bv.x PMC2230754

[55] Lofts H. Speciation of pyrithione in freshwaters [Internet]. CEH Project Number: C03634; 2009 [cited 2016 Feb 8]. http://nora.nerc.ac.uk/9924

[56] Marcheselli M, Rustichelli C, Mauri M. Novel antifouling agent zinc pyrithione: determination, acute toxicity, and bioaccumulation in marine mussels (*Mytilus galloprovincialis*). Environ Toxicol Chem. 2010 11;29(11):2583–92. 10.1002/etc.316 20853456

[57] Marcheselli M, Azzoni P, Mauri M. Novel antifouling agent-zinc pyrithione: Stress induction and genotoxicity to the marine mussel *Mytilus galloprovincialis*. Aquat Toxicol. 2011 3;102(1–2):39–47. 10.1016/j.aquatox.2010.12.015 21371611

[58] Ramu Y, Xu Y, Lu Z. Enzymatic activation of voltage-gated potassium channels. Nature. 2006 8 10;442(7103):696–9. 10.1038/nature04880 16799569

[59] Kurbannazarova RS, Bessonova SV, Okada Y, Sabirov RZ. Swelling-activated anion channels are essential for volume regulation of mouse thymocytes. Int J Mol Sci. 2011 12 8;12(12):9125–37. 10.3390/ijms12129125 22272123 PMC3257120

[60] Lewis SL, Ross PE, Cahalan MD. Chloride channels activated by osmotic stress in T lymphocytes. J Gen Physiol. 1993;101:801–26. 7687269 10.1085/jgp.101.6.801 PMC2216748

[61] Silva AL, Wright SH. Short-term cell volume regulation in *Mytilus californianus* gill. J Exp Biol. 1994 9;194:47–68. 7964405 10.1242/jeb.194.1.47

[62] Torre A, Trischitta F, Faggio C. Effect of CdCl2 on Regulatory Volume Decrease (RVD) in *Mytilus galloprovincialis* digestive cells. Toxicol In Vitro. 2013 6;27(4):1260–6. 10.1016/j.tiv.2013.02.017 23474061

[63] Thoroed SM, Fugelli K. The Na^(+)^-independent taurine influx in flounder erythrocytes and its association with the volume regulatory taurine efflux. J Exp Biol. 1994 1;186:245–68. 7525830 10.1242/jeb.186.1.245

