# Supplementary figures and images for: Osmoregulated Chloride Currents in Hemocytes from Mytilus galloprovincialis
Source: PLoS One. 2016 Dec 9;11(12):e0167972. doi: 10.1371/journal.pone.0167972 (PMC5148081; doi:10.1371/journal.pone.0167972)

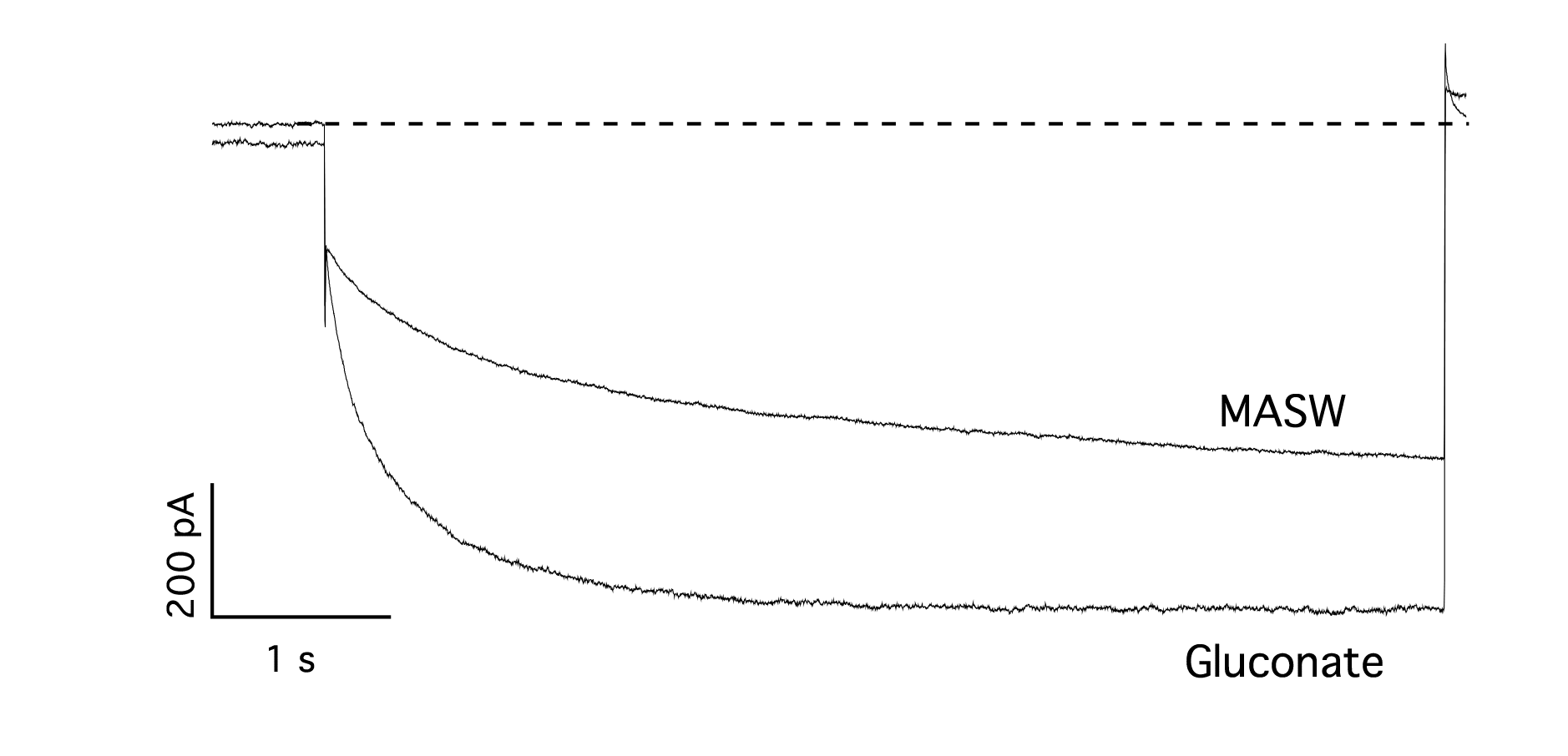

Supplement: S3 Fig — Currents recorded in MASW and in an identical solution where 460 mM NaCl in the bath was substituted by Na-Gluconate. Currents were elicited by a main pulse to -100 mV from a holding and tail voltages at V = +40 mV. (TIF) [file pone.0167972.s003.tif]
